# Two Antibiotics, Ampicillin and Tetracycline, Exert Different Effects in HT-29 Colorectal Adenocarcinoma Cells in Terms of Cell Viability and Migration Capacity

**DOI:** 10.3390/curroncol28040225

**Published:** 2021-07-04

**Authors:** Emil-Florin Hut, Matilda Radulescu, Nicolae Pilut, Ioana Macasoi, Delia Berceanu, Dorina Coricovac, Iulia Pinzaru, Octavian Cretu, Cristina Dehelean

**Affiliations:** 1 Faculty of Medicine, “Victor Babeș” University of Medicine and Pharmacy Timisoara, Eftimie Murgu Square No. 2, RO-300041 Timisoara, Romania; florin_hut@yahoo.com (E.-F.H.); radulescu.matilda@umft.ro (M.R.); pilut.ciprian@umft.ro (N.P.); tavi@octaviancretu.ro (O.C.); 2 Faculty of Pharmacy, “Victor Babeș” University of Medicine and Pharmacy Timisoara, Eftimie Murgu Square No. 2, RO-300041 Timisoara, Romania; dorinacoricovac@umft.ro (D.C.); iuliapinzaru@umft.ro (I.P.); cadehelean@umft.ro (C.D.); 3 Research Center for Pharmaco-Toxicological Evaluations, Faculty of Pharmacy, “Victor Babes” University of Medicine and Pharmacy Timisoara, Eftimie Murgu Square No. 2, RO-300041 Timisoara, Romania

**Keywords:** antibiotics, tetracycline, ampicillin, colorectal adenocarcinoma, HET-CAM assay, Hoechst staining

## Abstract

Antibiotics are considered the cornerstone of modern medicine; however, currently, antibiotic resistance has become a global health issue. Antibiotics also find new uses in the treatment of other pathologies as well as cancer. The present study aimed to verify the impact of tetracycline and ampicillin in a colorectal adenocarcinoma cell line, HT-29. The effects of the two antibiotics on cell viability and nucleus were evaluated by the means of MTT assay and the Hoechst staining method, respectively. The irritant potential at vascular level of the chorioallantoic membrane was tested by the HET-CAM assay. Treatment of HT-29 cells with the two antibiotics determined different effects: (i) tetracycline induced a dose- and time-dependent cytotoxic effect characterized by decreased cell viability, changes in cells morphology, apoptotic features (nuclear fragmentation), and inhibition of cellular migration, whereas (ii) ampicillin exerted a biphasic response—cytotoxic at low doses and proliferative at high concentrations. In terms of effect on blood vessels, both antibiotics exerted a mild irritant effect. These results are promising and could be considered as starting point for further in vitro studies to define the molecular mechanisms involved in the cytotoxic/proliferative effects.

## 1. Introduction

Despite the advancement of the research in the field of cancer, this disease remains a challenge for the global health system, causing each year millions of deaths worldwide. The conventional therapy for cancer includes chemotherapy, radiation therapy, and surgery, but the disadvantages related to these therapies, as severe side effects and low success rates, give enough room for the development of novel or repurposed therapies with increased efficiency and reduced adverse effects [1]. According to GLOBOCAN report 2020, colorectal cancer accounts for an estimated 9.4% of total deaths globally, occupying the second position as leading cause of cancer-associated death after lung cancer (an estimated 1.8 million deaths—18% of global number) [2]. Although the incidence of this type of cancer is constantly decreasing, an increase in disease diagnostic among the population up to 50 years has recently been recorded [3]. For this reason, colorectal cancer is considered a global health problem and many studies have focused on finding its promoting factors. There appears to be a direct correlation between gut microbes and tumor cell development [4]. The link between colorectal cancer and antibiotics use has been documented in numerous studies, showing that the risk of developing this type of cancer depends on the class of antibiotics used [5,6]. The mechanism underlying colorectal cancer appears to be the antibiotic-induced dismicrobism. Thus, broad-spectrum antibiotics act on the intestinal microbiota in the long run and facilitate colonization with pathogenic bacteria compared to narrow-spectrum antibiotics that do not have such a pronounced effect on the microbiota [7].

The discovery of antibiotics symbolized a major step for modern medicine. These drugs are considered the cornerstone of modern medicine, helping to cure many pathologies of infectious causes [8]. Today’s medicine and recent studies have turned their attention to new uses of antibiotics. In addition to their bactericidal or bacteriostatic effect, many antibiotics have found their use in cancer therapy. Numerous antibiotics with antitumor effects, such as adriamycin, bleomycin, or epirubicin, are already known and used in therapy. The mechanisms underlying the use of antibiotics as antitumor therapy are based on their ability to inhibit cell proliferation and to exert pro-apoptotic and anti-epithelial-to-mesenchymal transition effects [9]. 

At present, the role of antibiotics in cancer development is still debatable, since there are data in the literature that suggest a potential correlation between antibiotics use and an increased risk of developing tumors, but this matter requires more studies to have a complete image [10,11]. The main mechanism related to antibiotics’ increased risk of developing tumors is the alteration of the microbiota. Microbiota plays an important role in both immune processes and absorption of nutrients [12]. The classes of antibiotics involved in the onset of dismicrobism and increased cancer risk are beta-lactams, cephalosporins, and fluoroquinolones [11,13]. 

Tetracycline is the parent compound of the class of antibiotics with the same name and exhibits a bacteriostatic effect. These substances were first isolated from the culture of *Streptomyces aureofaciens.* Tetracycline, together with its semisynthetic derivatives, has a broad spectrum of antibacterial action, being active on both Gram-positive and Gram-negative bacteria. The tetracycline’s antibacterial mechanism of action consists in the blockage of protein synthesis by binding to the 30S ribosomal subunit [14]. Studies focusing on the antitumor effects of tetracycline have discovered several mechanisms, including (i) inhibition of mitochondrial protein synthesis, (ii) angiogenesis arrest, and (iii) inhibition of matrix metalloproteinases [15]. Tetracycline and its semisynthetic and synthetic derivatives have been studied for their potential antitumor effect. In studies of various cancers, it has been observed that these compounds act on several matrix metalloproteinases [16]. In addition, the synthetic derivative of tetracycline, Col-3 (4-dedimethylaminosancycline), is currently in clinical trials in cancer patients [17]. 

Ampicillin, a broad-spectrum bactericide agent, is part of the beta-lactam group and has been used for over 50 years to treat infections caused by both Gram-positive and Gram-negative bacteria [18]. The antibacterial mechanism of action of ampicillin lies in the binding of penicillin-binding proteins followed by inhibition of the transpeptidation reaction and peptidoglycan synthesis and the death of the bacterial cell [19]. In terms of the antitumor effect of ampicillin, the studies are complex and contradictory. Several in vitro studies have shown that ampicillin in various combinations has an antitumor effect in colorectal cancer [20,21]. At the same time, in vivo studies on different types of cancer have shown that ampicillin consumption increases the risk of developing certain cancers such as breast cancer [22]. 

In the present study we aimed to test two of the most frequently used antibiotics (tetracycline and ampicillin) in terms of the antitumor effect on the colorectal adenocarcinoma cell line, HT-29. The effects of the two antibiotics were investigated regarding the mechanism of cell death and possible toxic and blood vessel modifying effects on the chorioallantoic membrane.

## 2. Materials and Methods

### 2.1. Cell Culture

The cell line selected for this study, HT-29 (ATCC^®^ HTB-38 ™)—human colorectal carcinoma—was acquired as a frozen item from the American Type Culture Collection (ATCC). For cells’ culture and growth, a specific culture medium was used—McCoy’s 5a modified medium (ATCC^®^ 30-2007 ™)—which was completed with 10% FBS (fetal bovine serum, Gibco) and a 1% penicillin/streptomycin mixture (Sigma Aldrich, Merck KGaA Darmstadt, Germany). The experiments were performed in accordance with the standard conditions for cell culture, as follows: incubation at 37 °C in 5% CO_2_ atmosphere.

### 2.2. Cell Viability Assay

Cell viability was determined by colorimetric microculture tetrazolium assay (MTT). Cells were cultured in 96-well plates using a 1 × 10^4^ cells/well/200 µL medium. After cell attachment, cells were treated with five different concentrations of tetracycline and ampicillin (10, 25, 50, 75, and 100 µM), both solubilized in DMSO. The same concentrations were tested for DMSO, the solvent used for test compounds solubilization. Cell viability was determined at three-time intervals (24, 48, and 72 h). A volume of 10 µL/well of 3- (4,5-dimethylthiazol-2-yl) -2,5-diphenyltetrazolium bromide (MTT) solution (5 mg/mL) was added within the wells after the treatment periods. The cells were incubated for 3 h with the MTT reagent, followed by the addition of the solubilization buffer (100 µL/well) and stored for 45 minutes at room temperature and in the dark. The absorbance values of the reduced MTT were measured by the means of a microplate reader at 570 nm (xMark Microplate Spectrophotometer, Bio-Rad). The experiments were conducted in triplicate and the results were expressed as cell viability percentage (%).

### 2.3. Cells’ Morphology Assessment

To determine the cytotoxic potential of tetracycline and ampicillin, a microscopic evaluation of cells’ morphology and shape was performed. Cells were observed under bright field illumination and photographed at 24, 48, and 72 h after treatment with tetracycline and ampicillin (10, 25, 50, 75, and 100 µM), by comparison with DMSO. The photos were taken using Cytation 1 (BioTek Instruments Inc., Winooski, VT, USA). The analysis of the images was performed by means of Gen5™microplate data collection and analysis software (BioTek Instruments Inc., Winooski, VT, USA).

### 2.4. Scratch Assay

To observe the effect of the two antibiotics on the migratory capacity of the colorectal adenocarcinoma cells, HT-29, the scratch assay technique was performed. Cells were cultured in 12-well plates in a number of 2 × 10^5^ HT-29 cells/well. After reaching a confluence of about 90%, a line was drawn in the middle of the cell layer using a pipette tip. Cells that detached during the before mentioned procedure were removed by washing with PBS. Following these procedures, the cells were treated with five different concentrations of tetracycline and ampicillin (10, 25, 50, 75, and 100 μM) solubilized in DMSO. The cells were immortalized at the time intervals of 0 and 24 h, and the images obtained were compared with the control cells (untreated cells). The images were taken using Cytation 1 (BioTek Instruments Inc., Winooski, VT, USA). The analysis of the images was performed by means of Gen5™microplate data collection and analysis software (BioTek Instruments Inc., Winooski, VT, USA). In order to calculate the percentage of migration, the formula previously described by Felice et al. [23] was applied:

$$Scratch\;closure\;rate\;=\;\left[\frac{A_{t_{0}}\;-\;A_{t}}{A_{t_{0}}}\right]\;*\;100$$

$$\begin{array}{l} A_{t_{0}}\;-\;scratch\;area\;at\;time\;0\\ A_{t}\;-\;scratch\;area\;at\;24\;h \end{array}$$

### 2.5. Nuclear Staining

The Hoechst 33342 staining assay was performed to observe the impact of tetracycline and ampicillin on the HT-29 cells’ nucleus in order to define the type of cell death induced by these antibiotics. The applied protocol followed the manufacturer’s instructions. Cells were cultured at 1 × 10^4^ /well in 96-well plates. After reaching a confluence of approximately 80–90%, the cells were treated with five different concentrations of ampicillin and tetracycline (10, 25, 50, 75, and 100 μM), and in parallel the same five concentrations were tested for DMSO. After 24 hours, the medium was removed and 100 μL of staining solution was added to each well diluted 1:2000 in PBS. After incubation for 10 minutes at room temperature and protected from light, the staining solution was removed and washed three times with PBS. The pictures were taken with Cytation 1 (BioTek Instruments Inc., Winooski, VT, USA). The analysis of the images was performed by means of Gen5™microplate data collection and analysis software (BioTek Instruments Inc., Winooski, VT, USA). As a positive control for apoptosis induction, it was used 5 μM staurosporine (incubation for 3 h at 37 °C) and for necrosis 0.5% Triton X-100 (incubation for 30 min at 37 °C).

### 2.6. In Ovo Irritant Potential Assessment by the Means of Chorioallantoic Membrane (HET-CAM) Assay

The chorioallantoic membrane assay (HET-CAM) is a commonly used in ovo technique to verify the potential toxicity and irritant effect of different compounds, including therapeutic agents. This assay was performed on fertilized eggs (*Gallus gallus domesticus*) acquired from a local farm. The experimental protocol consisted of the following steps: (1) desinfection of the eggs with 70% alcohol; (2) placement of the eggs in a horizontal position in the incubator at 37 °C; (3) extraction of 7–8 mL of albumen at incubation day 3 (to allow the observation of blood vessels), (4) cutting a window in the upper part of the egg—incubation day 4 which was covered with adhesive tape during the incubation period, and (5) incubation until the day of HET-CAM test—incubation day 9.

The HET-CAM assay was performed on the ninth day of incubation. The solutions (SDS, H_2_O, DMSO, tetracycline, and ampicillin) were applied in a volume of 500 µL/egg. Five eggs were used for each solution. The changes in the blood vessels aspect were observed with the Discovery 8 Stereomicroscope, Zeiss, Göttingen, Germany, and the photos were taken with Axio CAM 105 color, Zeiss. All images were processed using ImageJ v 1.50e software (U.S. National Institutes of Health, Bethesda, MD, USA).

A positive control—SDS 1%—and a negative control—water—were used to evaluate the irritant effect. Tetracycline, ampicillin, and DMSO were tested at 100 µM. The effects monitored for 5 minutes at the vascular level were: hemorrhage (H), vessel lysis (L), and coagulation and extra vascular (C). To determine the irritant effect, the analytical method of calculating the irritation score (IS) was applied using the following formula [24]:

$$IS=5\times \frac{301-H}{300}+7\times \frac{301-L}{300}+9\times \frac{301-C}{300}$$

## 3. Results

### 3.1. Tetracycline Affects Cell Viability in a Dose and Time-Dependent Manner

**Figure 1:**
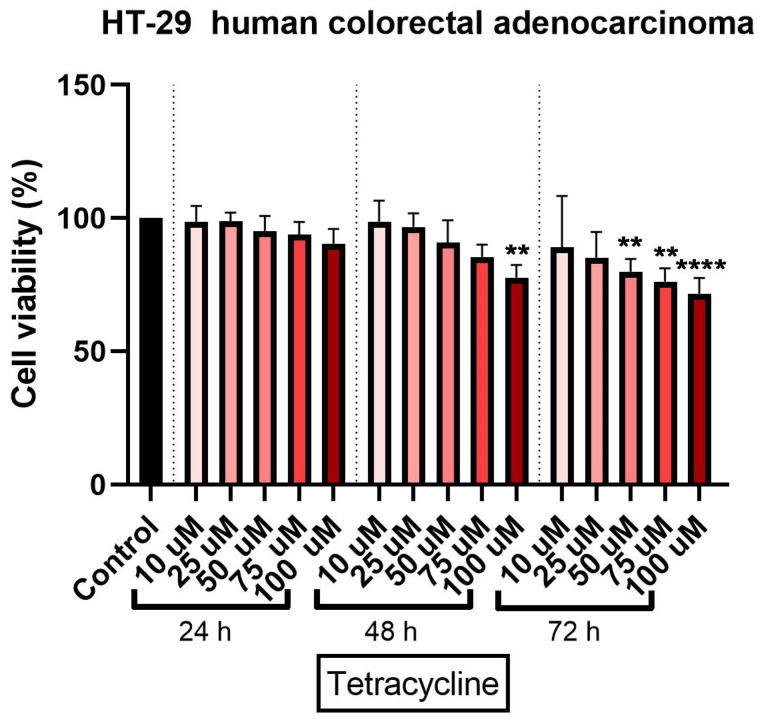
In vitro assessment of tetracycline’s (10, 25, 50, 75, and 100 µM) effect on cell viability in human colorectal adenocarcinoma (HT-29) cells. The results are expressed as cell viability percentage (%) normalized to control cells and were determined by MTT assay at 24-, 48-, and 72-h intervals. The data represent the mean values ± SD of three independent experiments performed in triplicate. One-way ANOVA analysis was applied to determine the statistical differences in relation to control followed by Dunnett’s multiple post-test comparisons (** *p* < 0.005 and **** *p* < 0.0001).

Tetracycline treatment exerted a concentration- and time-dependent cytotoxic effect in HT-29 cells. At 24 h, a relatively small decrease in cell viability was observed. The strongest cytotoxic effect was calculated at the highest concentration tested - 100 μM (approximately 90.4%) (Figure 1).

A 48-h treatment with tetracycline determined a more pronounced cytotoxic effect on the colorectal adenocarcinoma cells. As in the case of the 24-h exposure, a significant decrease in cell viability percentage was recorded, at the highest concentration (approximately 77%) (Figure 1).

The longest treatment with tetracycline—72 h—was associated with a decrease of cell viability percentage even at the lowest concentration tested—10 µM (approximately 86%). However, the most significant reduction of HT-29 cells viability percentage was recorded at the highest tested concentration of 100 µM a viability of approximately 71% (Figure 1). DMSO treatment at all three intervals (24, 48, and 72 h) did not significantly influence the cell viability percentage as compared to control cells (untreated cells) and this was the reason for normalizing the data to control.

### 3.2. Ampicillin Interfered Dose- and Time-Dependent in HT-29 Cells’ Viability

**Figure 2:**
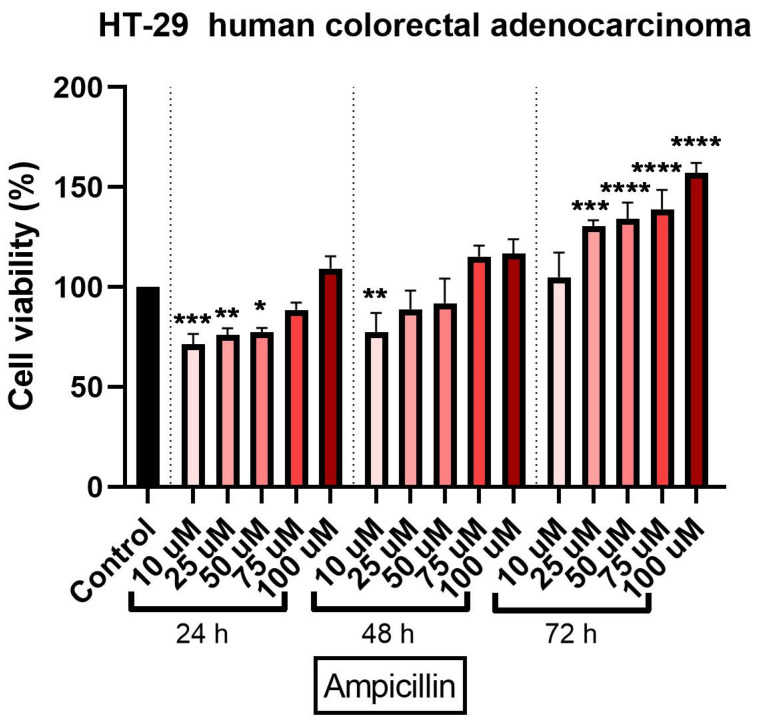
In vitro assessment of ampicillin’s (10, 25, 50, 75, and 100 µM) effect on cell viability in human colorectal adenocarcinoma (HT-29) cells. The results are expressed as cell viability percentage (%) normalized to control cells and were determined by MTT assay at 24-, 48-, and 72-h intervals. The data represent the mean values ± SD of three independent experiments performed in triplicate. One-way ANOVA analysis was applied to determine the statistical differences in relation to con-trol followed by Dunnett’s multiple post-test comparisons (* *p* < 0.05, ** *p* < 0.005, *** *p* < 0.001, and **** *p* < 0.0001).

The cytotoxic effect of ampicillin in HT-29 cells was verified by treating the cells with five concentrations (10, 25, 50, 75, and 100 µM) at three-time intervals (24, 48, and 72 h). An interesting finding was noticed in ampicillin-treated cells as compared to tetracycline ones: the lowest concentration—10 µM—induced the most significant decrease of cell viability percentage at all three intervals (73%, 72%, and 90%, respectively), whereas in the case of the other concentrations it was observed that by increasing the dose, the percentage of viable cells was higher (Figure 2). The most significant increase in cell viability percentage was recorded at 72 h post-treatment at the highest concentration tested (Figure 2).

### 3.3. Tetracycline and Ampicillin Treatment Determined Morphological and Cell Shape Changes in HT-29 Cells

**Figure 3:**
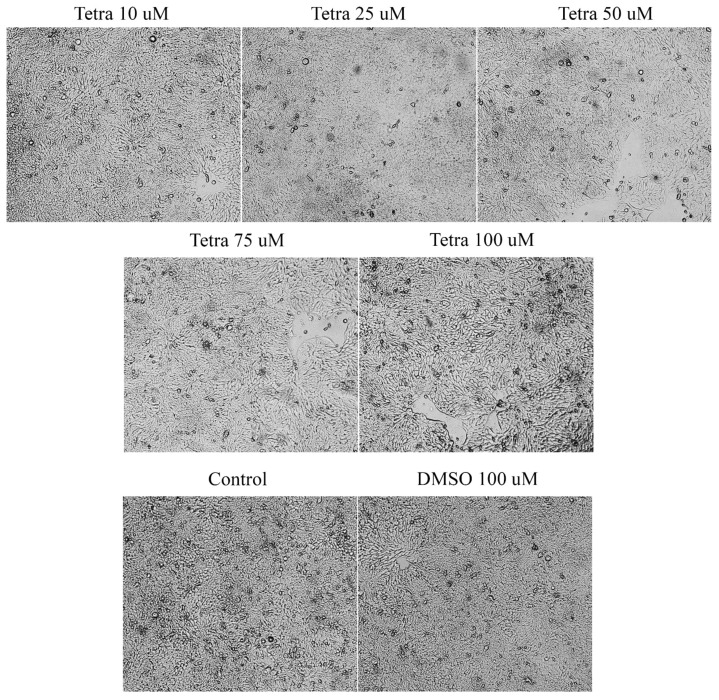
Morphological and shape changes produced by tetracycline treatment in HT-29 after 72 h of treatment. 20× magnification.

To evaluate the cytotoxic effect of tetracycline, the morphological and structural changes induced in the HT-29 cells were monitored at 24, 48, and 72 h after application. After 24 and 48 h, slight changes were observed in terms of cell morphology. However, the most visible changes were detected after the 72-h treatment (Figure 3), as follows: (i) The lowest concentrations tested (10 and 25 µM) caused small changes in cells’ structure and shape, as well as slight modifications in the confluence and adherence capacity of the cells; (ii) at 50 µM, several round and floating cells were observed; and (iii) the highest concentrations tested (75 and 100 µM) induced the most visible changes in shape and morphology together with a decrease in cell confluence compared to control cells and those stimulated with DMSO (Figure 3). In the case of the solvent (DMSO), no morphological changes were observed compared to the control cells (not stimulated).

**Figure 4:**
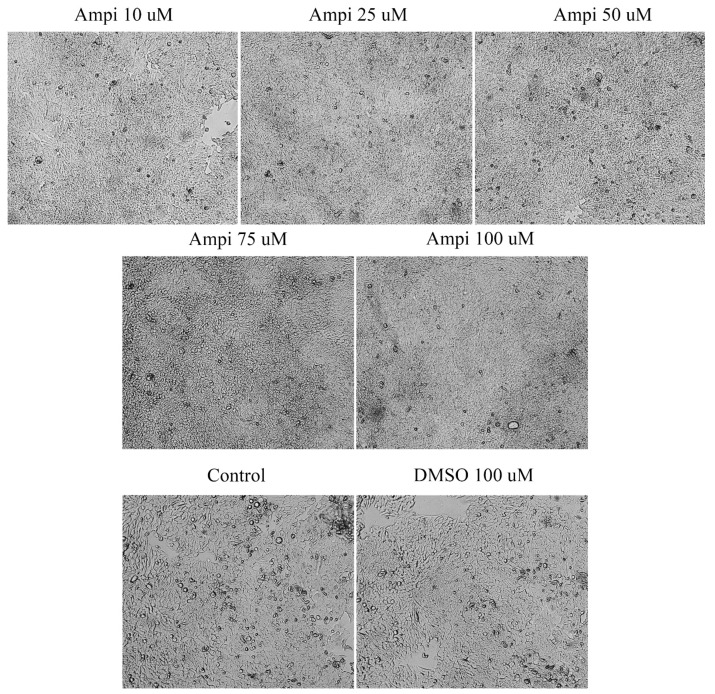
Morphological and shape changes produced by ampicillin in HT-29 after 72 h of treatment. 20× magnification.

In the case of ampicillin, a 72-h stimulation induced changes in terms of confluence and cells’ shape. At the lowest concentrations (10, 25, and 50 μM), free places were observed on the plate, the cells had a more roundish shape, showing signs of apoptosis. In the case of the two higher concentrations (75 and 100 μM) an increase in cell confluence was noticed. The cells occupied the entire plate indicating a high confluence and adherence capacity. In the case of the solvent, no major changes of cell morphology and confluence were observed (Figure 4). These data support the data obtained from the cell viability test.

### 3.4. Tetracycline and Ampicillin Influenced Cell Migration

**Figure 5:**
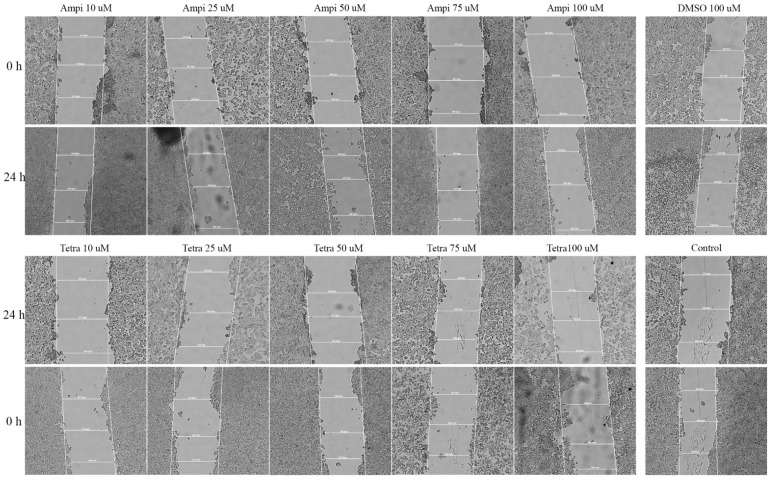
Representative images of the HT-29 scratch assay. Images were captured at 0 and 24 h after stimulation with tetracycline and ampicillin versus control cells. 20× magnification.

**Figure 6:**
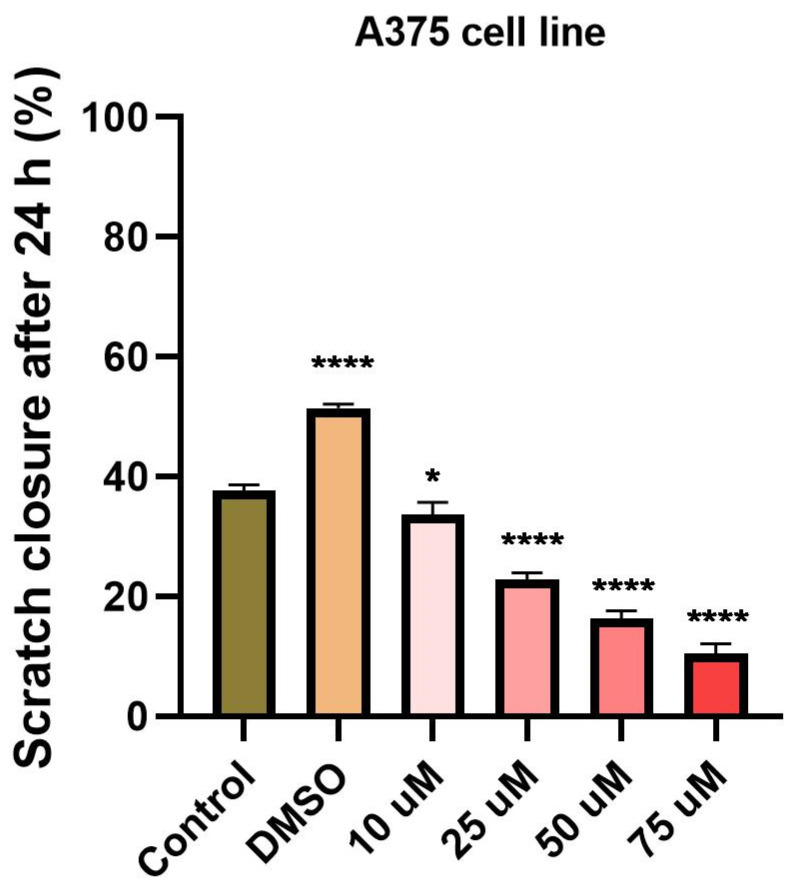
Scratch closure rate (%) following ampicillin and tetracycline treatment (10, 25, 50, 75, 100 µM) measured in HT-29 cells. The graph expresses the percentage of scratch closure after 24 h compared to the initial surface. The comparison between-groups was performed using One-way ANOVA test followed by Dunnett’s post-test (* *p* < 0.05; **** *p* < 0.0001 vs. control-cells).

To determine the effect of ampicillin and tetracycline on cell migration, a scratch assay was performed. Five concentrations of each antibiotic (10, 25, 50, 75, and 100 μM) were tested and compared with the effect of 100 μM DMSO and control cells that were not stimulated. In the case of ampicillin, a stimulation of cell migration was noted, being directly proportional to the tested concentration (Figure 5). In the case of the lowest concentration tested—10 μM—a 12.65% closure rate was calculated compared to the control group which presented a 23.28% rate. At the concentrations of 25, 50, and 75 μM, the following rates were calculated: 15.41%, 25.46%, and 28.19% (Figure 6). At the highest concentration tested—100 μM—a rate of 28.37% was obtained. Regarding the effect of tetracycline, it an opposite trend was noticed compared to that produced by ampicillin. At the concentrations of 10 and 25 μM rates of 17.27% and 17.12% were registered, respectively. The concentration of 50 μM induced a rate like that of the control group, of 22.71%. At the highest concentration tested, the closure rate decreased to 3.60%. In addition, changes in cell morphology were observed (Figure 5).

### 3.5. Tetracycline and Ampicillin Induced Apoptotic-Like Features in the Nucleus of HT-29 Cells

**Figure 7:**
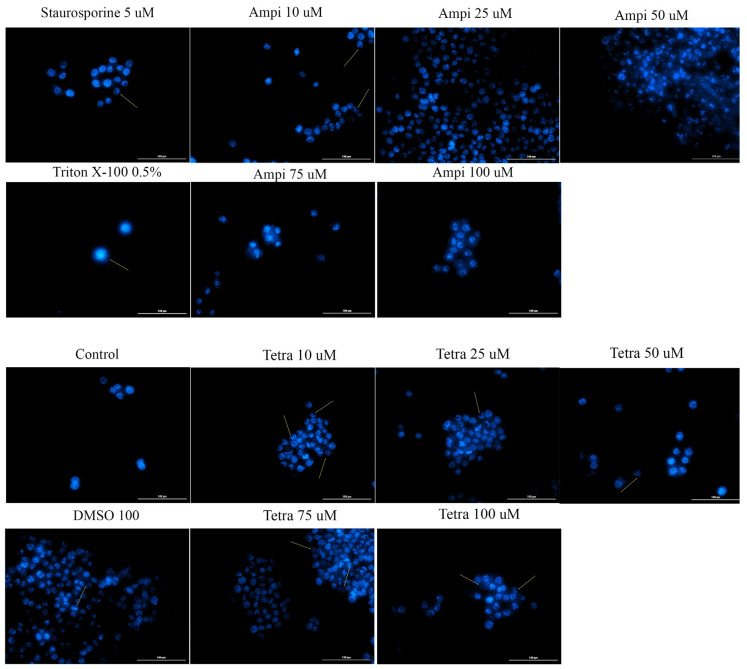
Cell nuclei staining using Hoechst 33342 in HT-29 cells after treatment with ampicillin and tetracycline (10, 25, 50, 75, and 100 μM) and DMSO for 24 h. The pictures were taken at 24 h post-treatment. Staurosporine solution (5 μM) represents the positive control for apoptotic changes and Triton X-100 solution (0.5%) for necrosis. The scale bar was 20 μm.

Changes in the nucleus may provide additional data on the cytotoxic effect of antibiotics by offering new insights concerning the type of cell death induced. To determine whether cell death occurred by apoptosis or necrosis, a Hoechst staining assay was applied. HT-29 cells were stimulated for 24 h with five different concentrations of ampicillin and tetracycline (10, 25, 50, 75, and 100 μM), and the results were compared with control cells, unstimulated, and stimulated cells with the solvent DMSO. In the case of the control and DMSO groups, it was observed that the nucleus of the cells had a round and regular shape, without signs of cell fragmentation. In the case of cells treated with tetracycline, a change in the shape of the nuclei was detected. They became condensed and numerous nuclear fragments were present, especially in the case of the highest concentration tested (100 μM). In the case of ampicillin, changes in the nuclei were minimal, being more obvious at the lowest concentration tested (Figure 7).

### 3.6. Tetracycline and Ampicillin Exerted a Slight Irritant Effect in Ovo

**Figure 8:**
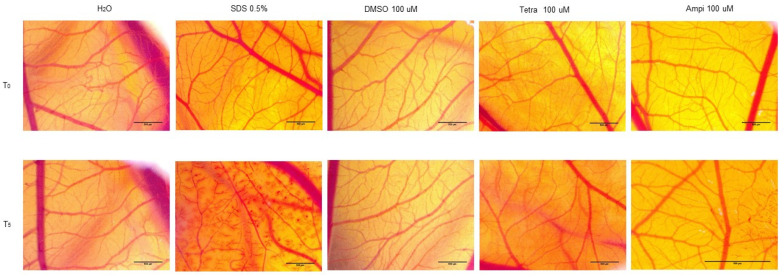
Representative stereomicroscopic images of CAMs (chorioallantoic membranes) treated with SDS—positive control, H_2_O; negative control, DMSO, ampicillin, and tetracycline. T_0_ represents 0 min after application of the test compounds and T_5_ represents 5 minutes after application of the compounds. Scale bar was 500 µM.

To verify the toxic and irritant potential of tetracycline, ampicillin, and DMSO, they were tested at the highest concentration (100 µM) in ovo, using chicken chorioallantoic membrane as a biological medium. The effects induced by tetracycline, ampicillin, and DMSO, together with the positive control (sodium dodecyl sulfate (SDS)) and the negative control (water) were assessed in the form of photographs at intervals of 0 minutes (before application) and at 5 min after application on the membrane. Three parameters were tracked: hemorrhage, lysis, and coagulation of blood vessels (Figure 8). These effects were evident only in the case of SDS, while in the case of water they were not observed. DMSO produced a slight vascular stasis at about two minutes after application. In the case of tetracycline, the noted effects were vascular lysis at about one minute after application and vascular stasis at approximately 4 minutes after application (Table 1). The egg specimens were verified for viability after test compounds treatment and the following results were obtained: (i) SDS induced the death of the specimens at one hour after application; (ii) after ampicillin application, the egg survived 24 h; and (iii) in the case of tetracycline, the egg was viable at 72 h after application. Tetracycline treatment exhibited a weak irritating effect, whereas in the case of ampicillin, the irritating effect was stronger.

## 4. Discussion

Colorectal cancer is ranked among the leading causes of cancer-related deaths worldwide [2]. The underlying risk factors of this type of cancer include lifestyle (smoking, obesity, reduced physical exercise), dietary habits (high intake of animal fat, red and processed meat, and low quantity of fibers), and age. An important role in the development of colorectal cancer was assigned in recent years to the colonic microbiota [5]. Alterations of the microbiota cause inflammation and promote the development of tumor cells [25]. Among the factors that could provoke a microbiotal dysbiosis could be mentioned the use of antibiotics [5]. The main classes of antibiotics involved in the development of dismicrobism are broad-spectrum antibiotics [26]. Several epidemiological studies have analyzed the correlation between the frequent use of antibiotics and the risk of colon cancer development, but the findings remain conflicting, mainly concerning the classes of antibiotics incriminated [5,13,27,28].

In the light of these data, the present study aimed to evaluate the potential toxic effects of two broad-spectrum and frequently prescribed antibiotics, tetracycline, and ampicillin, respectively, in vitro, using the colorectal adenocarcinoma cells (HT-29) as experimental model to assess their impact on cell viability and morphology, and migratory capacity and in ovo by applying HET-CAM assay for the evaluation of the irritant potential at vascular level. The selection of HT-29 cells as in vitro experimental model for the present study was based on the following arguments: (1) HT-29 cells represent one of the most common 2D in vitro models used in the studies of colorectal carcinoma [29]; (2) these cells have the capacity to keep their cellular properties unaltered even after 100 passages in culture [30]; and (3) HT-29 cells present a complex mutational status characterized by chromosomal instability phenotype and harbor multiple mutated genes with critical roles in cell proliferation, survival, and differentiation that define the oncogenic potential, the aggressiveness of the cancer cells, as well as the response to treatment, genes as *KRAS* wild-type, *BRAF, PIK3CA*, and *TP53* [29,31,32].

At present, the role of antibiotics in the field of cancer is uncertain due to the conflicting data available, data that on one hand present antibiotics linked with the risk of cancer [5,13,27] and on the other hand as anticancer agents [21,33]. Recent studies showed that the tetracycline class compounds have a potential antitumor effect in addition to the already known antibacterial effect. The semisynthetic derivative of tetracycline, doxycycline, has been studied on different types of tumor cells proving its inhibitory effect on their growth [33]. In addition, other synthetic tetracycline derivatives are currently in preclinical and clinical studies for their antitumor effect [34]. To date, it is known that the antitumor action of tetracyclines is based on several mechanisms, including: (i) inhibition of angiogenesis; (ii) inhibition of mitochondrial protein synthesis; (iii) inhibition of matrix metalloproteinases; and (iv) eradication of cancer stem cells [15]. For this reason, we aimed to test the parent compound of this class of antibiotics in terms of antitumor effect on the colorectal adenocarcinoma cell line. Our results showed that tetracycline induced a dose- and time-dependent cytotoxic effect characterized by a decrease of HT-29 cells’ viability percentage (the most significant effects being recorded after 72 h of treatment at the highest concentration tested—100 μM—Figure 1), changes in cells’ shape and morphology (round and detached cells and a reduced confluence, Figure 3), and apoptotic features (nuclear condensation and fragmentation, Figure 7). Similar results were presented by Ononda and colleagues that verified the effects of other tetracycline derivatives in the HT-29 cells. They observed that doxycycline and the tetracycline derivative, 3; 6-demethyl, 6-deoxy, 4-dedimethylamino tetracycline, had a dose- and time-dependent apoptotic effect [35], data that are in agreement with our results. Fife and colleagues tested doxycycline on a human osteosarcoma cell line and found that it had an inhibitory effect on proliferation causing cellular apoptosis [33]. As regards the antitumor effect of tetracycline derivatives, Iwasaki et al. demonstrated that doxycycline has an antiproliferative effect on human T-lymphoblastic leukemia CCRF-CEM cells by inhibiting the metalloproteinases and inducing cell apoptosis via activation of caspase-3 [16]. In another study performed on the colorectal adenocarcinoma cell line, HT-29, to compare the effect of doxycycline with cisplatin and oxaliplatin, it was observed that the tetracycline derivative activates caspase-3 in a manner similar to classical chemotherapeutics [36].

Ampicillin was also tested in the present study in terms of cytotoxic potential. Our data showed a different behavior of HT-29 cells in response to ampicillin treatment as compared to tetracycline, as follows: the lowest doses of ampicillin (10 and 25 µM) proved to be cytotoxic reducing cells’ viability (Figure 2), changing cells’ morphology (Figure 4), and inducing apoptotic features (Figure 7), whereas the highest concentrations (75 and 100 µM) exerted a stimulatory effect the cell viability being over 150% compared to the control cells. The effect of ampicillin could be considered a hormetic response. Hormesis can be defined as a biphasic dose-response phenomenon characterized by stimulatory of inhibitory effects at reduced doses that become reversed at high doses [37]. This feature of ampicillin has been shown in microbiology studies [38] and in plants [39], but its effect in vitro on tumor cell cultures has not been documented. In contrast to the studies that assert the role of ampicillin in the development of cancer, especially colorectal cancer, there are studies in the literature that highlight the antitumor effect of ampicillin administered under various formulations. The study by Ferraz et al. tested ampicillin salts as ionic liquids. They observed that under this formulation, ampicillin has an antiproliferative effect on five cancers, including colon cancer [21].

The opposite/antagonistic effects of tetracycline and ampicillin in HT-29 cells described in the present study are supported by the findings of several epidemiological study that identified an increased risk of colon cancer in patients that frequently used penicillins (particularly ampicillin/amoxicillin) as compared to those under treatment with tetracycline [5,13,27]. In another study performed on murine models, it was observed that the administration of a cocktail of antibiotics, including ampicillin, increases the risk of developing colorectal cancer compared to the control group [40].

Another aspect verified in the present study was the impact of the two antibiotics on HT-29 cells migratory capacity, a feature with critical role in cancer progression. Our results indicated that tetracycline treatment determined a dose-dependent inhibition of HT-29 cells migration, whereas in the case of ampicillin an inhibitory effect of observed only at low doses and a stimulatory one at high doses (Figure 5 and Figure 6), these data being correlated with the ones obtained for cell viability assessment. The data available concerning the effect of tetracycline/ampicillin on colorectal cancer cells migration are rather scarce. Several studies conducted on tetracycline derivatives proved an inhibitory effect of breast cancer [41] and non-small lung cancer cells’ migration [42].

As regards the irritant potential assessment performed by the means of HET-CAM assay, tetracycline showed a mild irritating effect (Figure 8 and Table 1). In a similar study performed in vitro, was shown that tetracycline derivatives had an inhibitory effect on angiogenesis, this aspect being important regarding their possible anti-metastatic effect [43]. As regards the ampicillin, its irritating effect at vascular level was stronger as compared to tetracycline (Figure 8 and Table 1). This aspect is important in terms of the ability of ampicillin to influence the formation of blood vessels and its effect on changes regarding the bleeding, lysis and coagulation because these parameters can interfere with the kinetics of the antibiotic in the body. In a study by Wu et al., it was shown that in addition to the influence of the microbiota exerted by antibiotics, including ampicillin, they can also influence angiogenesis and other changes in blood vessels architecture in a murine model, on blood vessels in the cornea [44].

## 5. Conclusions

Our present work offers details regarding the behavior of HT-29 colorectal adenocarcinoma cells after treatment with tetracycline and ampicillin in terms of cells’ viability, morphology, and migration capacity suggesting different effects of the two compounds, as follows: (i) a dose- and time-dependent cytotoxic effect for tetracycline and (ii) a hormetic-like effect for ampicillin—cytotoxic at low doses and proliferative at high doses. In addition, the two antibiotics exerted an irritating effect at vascular level of chorioallantoic chick membrane. These data could be considered a reliable background for further in vitro and in vivo studies that will investigate the molecular mechanisms underlying the cytotoxic/pro-tumoral potential of the two antibiotics analyzed in this study.

## Figures and Tables

**Table 1:** Irritation score (IS) for SDS (sodium dodecyl sulfate), H_2_O, DMSO, ampicillin, and tetracycline (100 µM) and the occurrence time of hemorrhage (tH), lysis (tL), and coagulation (tC).

	SDS 0.5%	H_2_O	DMSO 100 µM	Tetra 100 µM	Ampi 100 µM
IS	19.24	0.07	4.57	7.36	9.16
tH	15 s	300	300	300	202
tL	20 s	300	300	71	137
tC	37 s	300	150	235	178

## Data Availability

The data presented in this study are available on request from the corresponding author.
